# Clinical Performance Tradeoffs of ChatGPT-5.2 Thinking (OpenAI) Compared with Radiologist Interpretation in Biopsy-Referred Mammography: Cancer Detection, False Positives, and Laterality

**DOI:** 10.3390/tomography12040045

**Published:** 2026-03-29

**Authors:** Mohammad Alarifi, Areej Aloufi, Abdulrahman Jabour, Ahmad Abanomy, Haitham Alahmad, Khaled Alenazi, Alhanouf Alshedi, Mansour Almanaa

**Affiliations:** 1 Radiological Sciences Department, College of Applied Medical Sciences, King Saud University, P.O. Box 145111, Riyadh 4545, Saudi Arabia; malarifi@uwm.edu (M.A.); aaloufi@ksu.edu.sa (A.A.); aabanmi@ksu.edu.sa (A.A.); hnalahmad@ksu.edu.sa (H.A.); kenazi@ksu.edu.sa (K.A.); aalshedi@ksu.edu.sa (A.A.); 2 Visiting Scholar, Department of Health Informatics & Administration, University of Wisconsin–Milwaukee, Milwaukee, WI 53201, USA; 3 Department of Public Health, Faculty of Nursing and Health Science, Jazan University, P.O. Box 114, Jazan 45142, Saudi Arabia; ajabour@jazanu.edu.sa

**Keywords:** artificial intelligence, mammography, breast cancer, BI-RADS, diagnostic accuracy, reader study

## Abstract

**Background/Objectives:** Breast cancer screening such as mammography supports earlier detection, but variability in interpretation can still lead to missed cancers and avoidable follow-up testing. We evaluated ChatGPT-5.2 Thinking (OpenAI) as a stand-alone model for examination-level malignancy classification on standard bilateral mammography views in a biopsy-referred cohort, compared with breast radiologists, and assessed laterality performance. **Methods:** We conducted a retrospective, multicenter diagnostic-accuracy study across breast imaging centers in Saudi Arabia. From an upstream screened cohort (*n* = 1225), we constructed a biopsy-referred test set of 100 mammography examinations (four 2D views per exam: bilateral CC and MLO; 400 images), including 61 biopsy-confirmed malignancies and 39 biopsy-negative controls, with pathology as the reference standard. Radiologists were blinded to pathology and AI outputs and assigned BI-RADS (0–5) and suspected laterality. ChatGPT-5.2 interpreted the same de-identified views using a BI-RADS-guided prompt to generate BI-RADS and laterality. The sensitivity, specificity, accuracy, and laterality concordance were then estimated. **Results:** ChatGPT-5.2 had higher sensitivity than radiologists (95.08% vs. 81.97%) but markedly lower specificity (10.26% vs. 56.41%), resulting in lower overall accuracy (62.00% vs. 72.00%). The AI produced 58 true positives, 35 false positives, and 3 false negatives, while radiologists produced 50 true positives, 17 false positives, and 11 false negatives. Laterality accuracy among malignant examinations was 60.66%. **Conclusions:** In this pathology-anchored, biopsy-referred evaluation, ChatGPT-5.2 identified more cancers but generated substantially more false-positive classifications and showed only moderate breast-side localization. These findings support use as a concurrent aid or prioritization tool rather than a stand-alone reader and motivate efforts to improve specificity and laterality before prospective validation.

## Simple Summary

Mammograms can help find breast cancer early, but readings can differ, which may lead to missed cancers or require additional tests that are not needed. This study compared an artificial intelligence program (ChatGPT-5.2 Thinking) with breast radiologists using standard mammogram images from people who later had a biopsy (a small tissue sample test) to confirm whether cancer was present. The program found more cancers, but it also flagged many non-cancer findings as suspicious and was only moderately accurate in choosing the correct breast. In practice, it may serve as a second check or help sort urgent cases, not replace radiologists.

## 1. Introduction

Breast cancer remains a leading cause of cancer-related morbidity and mortality among women worldwide and continues to place a substantial clinical and economic burden on health systems [1,2]. Screening mammography has been central to early detection programs and has contributed to stage migration and improvements in survival through earlier diagnosis and timely intervention [3,4]. Despite these gains, the performance of screening remains variable across programs and settings. Reader sensitivity and specificity depend on multiple factors that include breast density, lesion conspicuity, workload, double-reading policies, training, and access to prior images for comparison [5,6,7,8]. False negatives may delay diagnosis while false positives may trigger unnecessary callbacks, added imaging, anxiety, and biopsies that do not yield cancer [9,10,11]. These realities have motivated sustained efforts to enhance accuracy while preserving efficiency in high-volume screening environments [12,13].

Artificial intelligence-based systems for mammography have emerged as a promising approach to support detection and decision-making [14,15]. Deep learning methods trained on large datasets can learn visual patterns associated with masses, microcalcifications, and asymmetries and then produce image-level or case-level predictions that can be integrated into the reading workflow [16,17]. Several studies report that AI can match or approach single-reader performance and can assist readers through triage, prioritization, or second reader-style support [18,19,20,21]. At the same time, the literature also documents failure modes that include difficulties with subtle calcifications, asymmetry, and laterality or localization errors, as well as performance variability across vendors, acquisition protocols, and clinical settings [22,23,24,25]. There is an ongoing need for rigorous evaluation against robust reference standards and for transparent reporting of metrics that matter to patients and clinicians [26,27]. In parallel, patients increasingly view their reports directly via portals, and a recent systematic review shows sustained patient demand for access alongside comprehension- and anxiety-related challenges; therefore, readability and patient-centered communication should follow explicit design criteria when integrating AI outputs into screening workflows [28]. In this context, patient-facing AI interpretations could function as a structured “second look” explanation that helps patients formulate questions and participate in shared decision-making during clinical consultations. However, such use should be positioned as decision support that complements rather than replaces radiologist assessment and clinician-guided management.

Biopsy confirmation offers a decisive reference to evaluate diagnostic systems because it anchors analyses to ground truth rather than to follow-up imaging alone [29]. Cohorts built on biopsy-confirmed cancers allow unbiased estimation of sensitivity and enable stratified analyses by lesion type, size, and breast density [29,30]. When paired with an appropriate sample of biopsy-negative controls or verified benign outcomes, these cohorts also enable meaningful estimation of specificity and accuracy [30]. In addition, expert review of discordant cases can reveal systematic error patterns that inform future model development, calibration, and deployment strategies [31].

Importantly, unlike dedicated mammography AI systems, which are typically developed using convolutional or related vision architectures trained specifically for breast imaging tasks, ChatGPT-5.2 Thinking represents a general multimodal large language model capable of image-informed reasoning across domains. Accordingly, the present study was designed to evaluate whether such a general reasoning model could provide clinically meaningful performance in biopsy-referred mammography, rather than to position it as equivalent to a purpose-built breast imaging AI system.

The present study evaluates ChatGPT-5.2 Thinking (OpenAI) as a stand-alone model for the clinically defined imaging task of examination-level malignancy classification using bilateral CC and MLO screening mammography views in a biopsy-referred cohort with pathology reference. This study aimed to compare the diagnostic performance of ChatGPT-5.2 Thinking with that of board-certified breast radiologists, using biopsy-confirmed pathology as the gold standard. In addition to case-level sensitivity, specificity, and accuracy, we assessed laterality performance and feature-type error patterns across four radiologic categories (mass, asymmetry, microcalcifications, and parenchymal distortion) to identify clinically actionable failure modes [32].

## 2. Materials and Methods

### 2.1. Design and Setting

A retrospective, multicenter diagnostic-accuracy study was conducted across several cities in Saudi Arabia within participating breast imaging centers. An upstream screening population of 1225 women was accrued from multiple health systems during the study period. From this screened cohort, we constructed a biopsy-referred test set by including examinations that proceeded to image-guided or surgical biopsy based on clinical assessment. The analytic test set comprised 100 biopsy-referred mammography examinations (four standard 2D views per exam; 400 images total), including 61 biopsy-confirmed malignancies and 39 biopsy-negative controls, linked deterministically to each site’s pathology database to establish a biopsy-anchored reference standard. Radiologist interpretations were based on the original finalized clinical mammography reports retrieved from the participating hospitals. In these institutions, mammography reports are reviewed and approved by consultant radiologists according to hospital policy; therefore, the radiologist reference used in this study reflects consultant-approved clinical interpretations rather than trainee-only reads. This study relied on the original approved report for each examination and did not use a separate research consensus rereading.

This design was selected to ensure definitive ground truth for malignancy status and to enable direct estimation of sensitivity and specificity against pathology. However, because the cohort is enriched for biopsy-referred cases, it is not representative of a general screening population and may yield inflated sensitivity and depressed specificity relative to true screening prevalence; we therefore interpret performance as applicable to a biopsy-referred triage/concurrent-aid scenario rather than population screening. Accordingly, we quantified case-level sensitivity, specificity, and accuracy for malignant vs. benign biopsy outcome (primary endpoint) and evaluated laterality performance and feature-type patterns (secondary endpoints).

### 2.2. Imaging and Readers

For each case, bilateral craniocaudal (CC) and mediolateral oblique (MLO) views were exported as DICOM files and de-identified; each case comprised four images. Board-certified breast radiologists were blinded to pathology and to ChatGPT-5.2 Thinking outputs. Radiologists provided study-level BI-RADS^®^ assessment categories (0–5) and documented the suspected laterality (right vs. left) of the abnormality prompting the assessment. Radiologist BI-RADS categories were used to generate binary calls at pre-specified thresholds for comparison with pathology and to classify true-positive, false-positive, true-negative, and false-negative outcomes. Laterality was used to define side-specific correctness among positive cases.

### 2.3. Prompt Setting and Session Standardization

**Figure 1:**
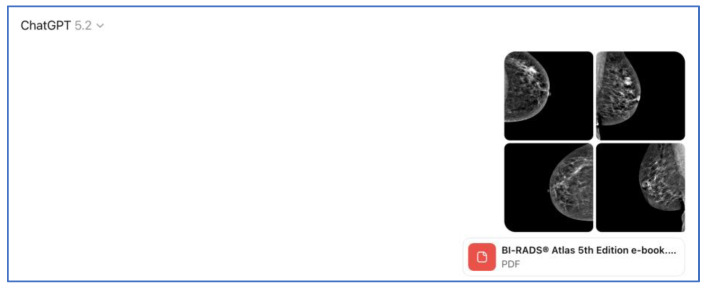
Example of the structured output generated by ChatGPT-5.2 Thinking for a breast imaging case using the study prompt.

**Figure 2:**
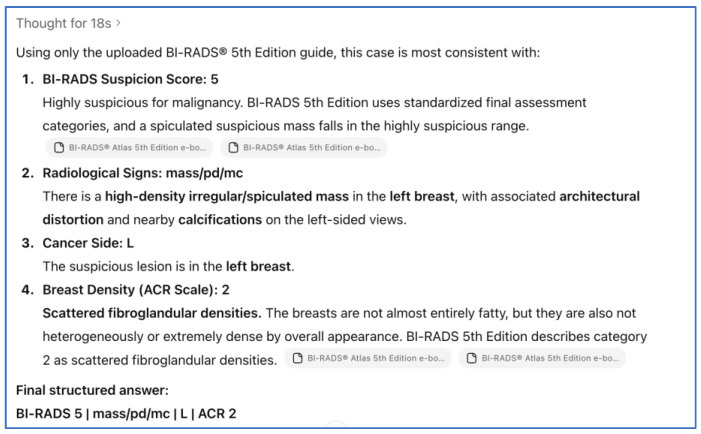
Example of structured case-level output from ChatGPT-5.2 Thinking.

**Figure 3:**
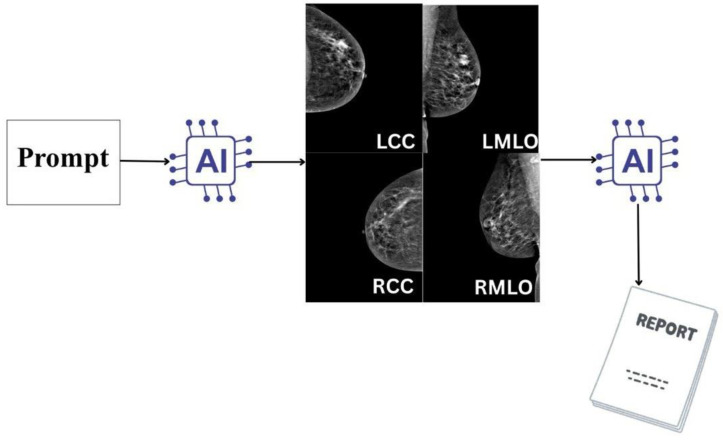
Prompting workflow used for evaluation of ChatGPT-5.2 Thinking on de-identified bilateral CC and MLO views, producing BI-RADS (0–5) and suspected laterality.

To improve transparency and reproducibility, the exact prompt used for AI interpretation is reported below. The same prompt was applied consistently for all case evaluations performed from 1 September 2025 onward. During prompt setup, expert radiologists Dr. AD and Dr. KA reviewed the wording and expected output structure to confirm that the prompt was clear, clinically relevant, and aligned with BI-RADS^®^ 5th Edition terminology (American College of Radiology, Reston, VA, USA). As part of prompt stability assessment, each case was evaluated in a new chat session to minimize contextual carryover between cases, a step taken to reduce the possibility that prior case content could influence subsequent interpretations through retained session context within the chatbot environment. The prompt and reading workflow were also tested across different user accounts and devices by two independent researchers to confirm consistent prompt behavior and output structure under the same instructions (Figure 1, Figure 2 and Figure 3) (Appendix A). We additionally recorded approximate AI inference time per case, defined as the elapsed time from case submission to complete response generation; across the 100 evaluated cases, the minimum runtime was 17.00 s, the maximum runtime was 21.00 s, and the average runtime was 18.79 s.

### 2.4. Artificial Intelligence and Prompting

ChatGPT-5.2 Thinking (OpenAI) was the only AI system evaluated in this study (Figure 3). The model was used in stand-alone mode to interpret de-identified bilateral CC and MLO mammography views. A fixed, pre-specified prompt instructed the model to produce the following: (i) an ordinal BI-RADS^®^ assessment category (0–5) using BI-RADS-consistent terminology, (ii) a binary malignancy classification derived from a pre-defined BI-RADS threshold, and (iii) suspected laterality (right vs. left) when an abnormality was identified. BI-RADS categories 1 and 2 were classified as negative, while BI-RADS categories 3, 4, and 5 were classified as positive. This threshold was applied consistently to both the radiologist and AI model assessments. Sensitivity, specificity, and overall diagnostic accuracy were subsequently calculated.

The prompt was finalized prior to evaluation and applied uniformly across cases. ChatGPT-5.2 Thinking was not used to generate ground-truth labels, alter images, select cases, or compute performance statistics; all metrics were calculated by the study team using pathology as the reference standard.

### 2.5. Statistical Analysis, Software, and Reproducibility

Categorical variables were summarized as counts and percentages and continuous variables as means with standard deviations or medians with interquartile ranges, according to distributional shape. Primary case-level performance metrics were sensitivity, specificity, and overall accuracy; laterality accuracy (right vs. left breast) was analyzed as a secondary endpoint. Exact 95% confidence intervals were calculated for proportions using the Clopper Pearson method, and paired proportions were compared with McNemar tests. For the purpose of performance calculation, BI-RADS categories 1 and 2 were classified as negative and BI-RADS categories 3, 4, and 5 as positive. This threshold was chosen to reflect a clinically actionable recall decision rather than discrimination across ordinal scores. Therefore, ROC curve, AUC, and calibration analyses were not performed. Instead, a forest plot was constructed to visually summarize and compare the 95% confidence intervals of key diagnostic performance metrics between the radiologist and ChatGPT-5.2. Missing data were handled with complete-case analysis. All hypothesis tests were two-sided with α = 0.05. Pre-specified subgroup analyses included lesion “sign” (mass, asymmetry, microcalcifications, and parenchymal distortion) and breast density categories when sample size permitted stable estimates.

All analyses were executed in Python and R on secured institutional workstations. Reproducible pipelines were implemented as version-controlled scripts with fixed random seeds, where applicable; summary tables and figures were generated directly from analysis outputs. Parameter settings, analysis logs, and software versions were archived alongside the study protocol to facilitate replication. Institutional oversight was provided by the University of Wisconsin–Milwaukee IRB (IRB #20.230), with a waiver of informed consent for the use of de-identified, minimal-risk data. Data availability: De-identified imaging data cannot be publicly shared due to patient-privacy restrictions and institutional policy. Analysis code resides on an access-controlled, encrypted institutional workstation under the corresponding author’s account and can be provided to qualified researchers upon reasonable request, subject to IRB oversight and a data-use agreement.

## 3. Results

### 3.1. Diagnostic Performance Comparison Between Radiologists and AI

**Figure 4:**
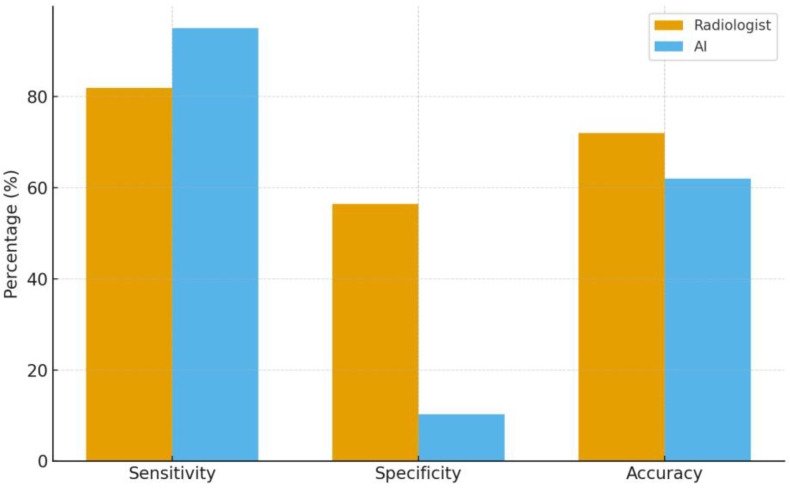
Diagnostic performance tradeoffs of AI versus radiologists in a biopsy-referred cohort (sensitivity, specificity, and accuracy).

**Figure 5:**
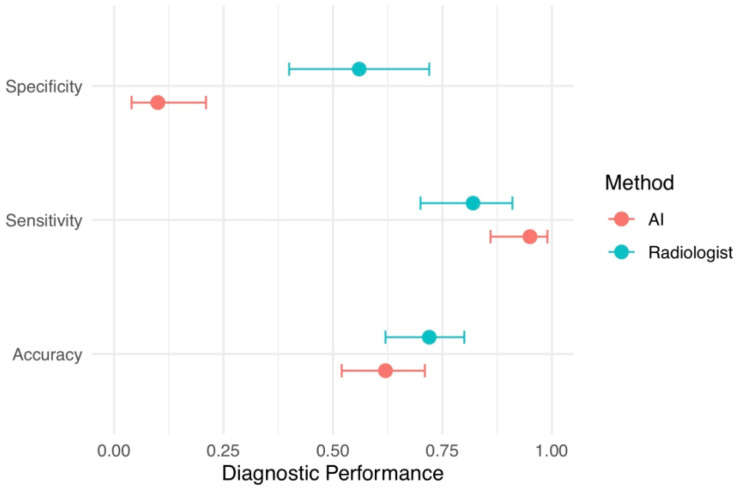
Forest-style plot comparing diagnostic performance of ChatGPT-5.2 Thinking and radiologists in the biopsy-referred cohort.

Figure 4 presents a comparative analysis of radiologist and AI diagnostic performance metrics, using biopsy results as the gold standard. The AI model demonstrated higher sensitivity than the radiologist for the detection of biopsy-confirmed malignancy at 95.1% (95% CI, 86.3–98.7%) versus 82.0% (95% CI, 70.0–91.0%), respectively. Specificity was lower for the AI model at 10.3% (95% CI, 4.0–21.0%) compared with 56.4% (95% CI, 40.0–72.2%) for the radiologist. Overall diagnostic accuracy was higher for the radiologist at 72.0% (95% CI, 62.1–80.5%) than for the AI model at 62.0% (95% CI, 52.0–71.4%) (see Figure 4 and Figure 5).

In absolute terms, ChatGPT-5.2 correctly identified 58 true-positive cases but also generated 35 false-positive predictions, while radiologists recorded 50 true positives and 17 false positives. The AI missed only three true malignancies (FN = 3), whereas radiologists missed eleven (FN = 11). Although AI demonstrated better sensitivity, expert image review revealed that many of its false-positive detections corresponded to benign structures or peripheral artifacts, indicating misclassification rather than true lesion recognition. The full classification results and diagnostic performance metrics are summarized in Table 1.

### 3.2. AI Performance by Radiologic Feature Type

**Figure 6:**
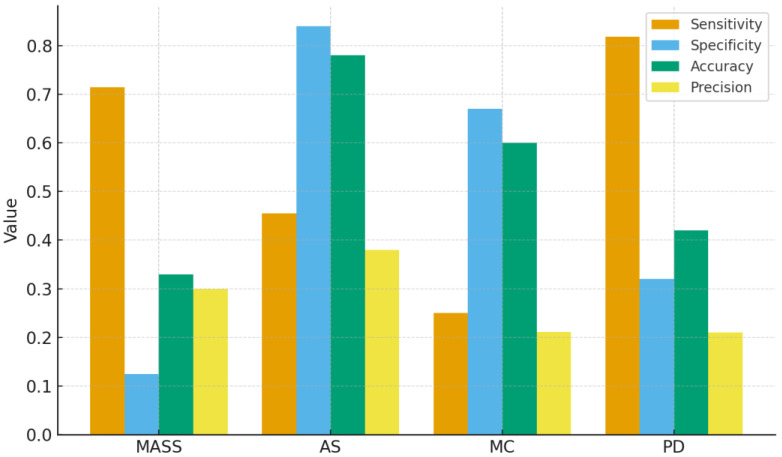
AI performance stratified by radiologic feature type (mass, asymmetry, microcalcifications, and parenchymal distortion).

Table 2 and Figure 6 summarize the AI model’s diagnostic metrics across four radiologic lesion categories: mass, asymmetry (AS), microcalcifications (MC), and parenchymal distortion (PD). Among all lesion types, parenchymal distortion (PD) achieved the highest sensitivity (0.818), suggesting that AI performed best when detecting diffuse density changes. Mass lesions also demonstrated relatively high sensitivity (0.714) but suffered from extremely low specificity (0.125), reflecting a tendency of AI to misclassify normal dense areas as suspicious masses. Asymmetry (AS) showed a balanced performance, with sensitivity of 0.455 and high specificity of 0.84, the most favorable trade-off among all categories. In contrast, microcalcifications (MC) were the weakest feature type for AI, with sensitivity of only 0.25 and precision of 0.211, indicating poor recognition of small-scale calcified foci. Taken together, these results suggest that AI performs relatively well in identifying large-area abnormalities (PD, mass) but lacks reliability in localizing fine-grained structures (MC, AS). This variability across lesion types is consistent with radiologists’ qualitative observations that AI often highlights regions adjacent to, rather than directly over, true lesions.

### 3.3. AI Performance in Breast-Side Localization

**Figure 7:**
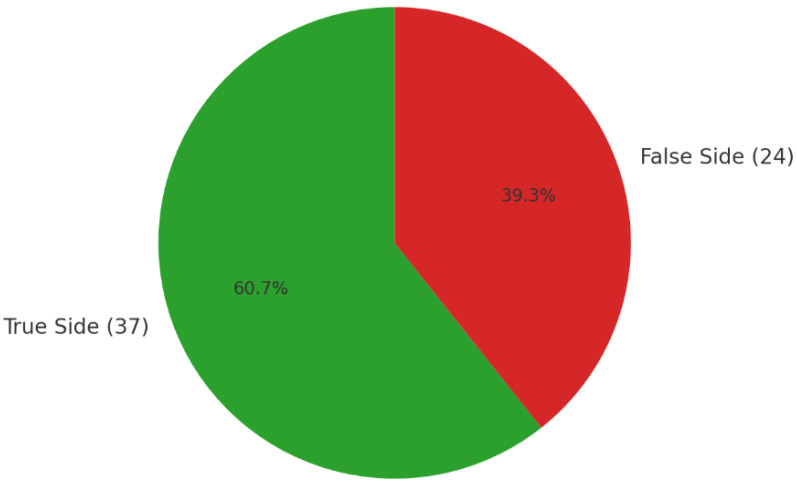
Laterality concordance of AI (right vs. left breast) among malignant examinations in the biopsy-referred cohort.

Figure 7 displays the AI’s accuracy in correctly identifying the laterality (right vs. left breast) of detected abnormalities. The model correctly localized the disease in 37 cases (“True side”) and failed in 24 cases (“False side”). This yields an overall laterality accuracy of 60.66%, reflecting moderate spatial awareness but insufficient precision for clinical reliability. In many incorrect cases, AI successfully detected the presence of a lesion but marked it on the contralateral breast or outside the actual parenchymal area. Such errors emphasize the model’s limitations in spatial alignment and contextual awareness, particularly in cases with overlapping tissue density or bilateral findings.

## 4. Discussion

In this biopsy-referred cohort with biopsy-confirmed diagnoses, the AI model demonstrated higher sensitivity but substantially lower specificity compared with radiologists, resulting in a significantly elevated false-positive rate. In the context of malignancy detection, sensitivity is generally regarded as the primary performance metric, as failure to identify a true malignancy risks delaying diagnosis and deferring potentially curative treatment. From this perspective, the superior sensitivity demonstrated by the AI model may confer a clinically meaningful advantage. Nevertheless, an optimal diagnostic test must balance sensitivity against an acceptable false-positive burden. Specificity and positive predictive value remain indispensable performance domains, as deficiencies in these metrics are associated with elevated rates of unnecessary recall, short-interval surveillance imaging, benign tissue sampling, patient psychological distress, and incremental health system burden [1,2]. Accordingly, the trade-off observed here may be acceptable only in a supportive or triage-oriented role where radiologists retain final interpretive authority, rather than as a stand-alone replacement for expert breast imaging assessment. In this context, the current results support the view that AI may assist radiologists in prioritizing suspicious examinations, but they do not eliminate the need for radiologists, especially when false-positive control, lesion localization, contextual judgment, and final clinical responsibility are required [8,16].

The observed performance pattern suggests a potential role for the AI model as a supplementary decision-support tool, whereby its high sensitivity may reduce the rate of missed malignancies in cases otherwise classified as benign by the radiologist. However, the substantially elevated false-positive rate identified in the present study indicates a risk of increased unnecessary recall and additional diagnostic investigations, with associated patient psychological burden. Furthermore, the integration of AI-generated assessments into clinical communication pathways may facilitate patient understanding of imaging findings and the rationale for further investigation, potentially supporting more informed clinical consultations. These potential benefits, however, require prospective validation before clinical implementation can be recommended. This tradeoff is consistent with prior evaluations where deep learning systems increase case finding but introduce benign overcalls under a real-world mix of screening and diagnostic studies [1,2,4,6,11,17,22,24]. Therefore, the most appropriate near-term clinical framing is triage/prioritization or concurrent-aid support, not replacement of radiologist interpretation [5,10,15,20].

Feature-level behavior clarifies where artificial intelligence helped and where it struggled. Parenchymal distortion produced the highest sensitivity, which aligns with studies showing that textural and global parenchymal patterns are well captured by convolutional and transformer-based encoders trained at image scale [7,13,18]. Asymmetry achieved the best specificity but only modest sensitivity, a conservative posture that mirrors prior reports in which models avoid distortion overcalls yet miss subtle tethering or spiculations in dense tissue [9,14,18]. Mass showed moderate sensitivity but very low specificity, consistent with the known risk of mistaking overlapping tissue and benign masses for malignancy, especially in heterogeneously dense breasts [3,5,19,29]. Microcalcifications remained the weakest category, echoing the literature that small, sparse, and high-frequency signals are sensitive to pixel resolution, preprocessing, and training set curation [8,12,23,27]. Together these patterns suggest practical parameterization: use conservative thresholds when calcifications or mass-like appearances are suspected, while allowing artificial intelligence more freedom to surface diffuse parenchymal abnormalities [10,13,18].

Laterality accuracy was 60.7%, which represents a meaningful constraint for workflow. Wrong side assignment can lead to additional views, unnecessary callbacks, and avoidable anxiety, even when the case is correctly classified as positive [3,16,26]. This laterality limitation is likely related to the model’s reduced ability to maintain spatial alignment and side-specific contextual awareness across bilateral CC and MLO views. Although the model often detected that an abnormality was present, it appeared less consistent in correctly mapping that finding to the right versus left breast across projections. This pattern suggests difficulty in preserving cross-view correspondence and examination-level right–left anatomical context, which may have contributed to contralateral assignment errors despite retained sensitivity for abnormality detection. Multiple groups have improved side awareness by enforcing cross-view consistency checks and by incorporating quadrant-aware attention maps or side-conditioned decoders [7,17,31]. These findings further suggest that a general multimodal reasoning model such as ChatGPT-5.2 Thinking should not be considered a stand-alone mammography interpretation tool at its current performance level but rather a supportive system requiring radiologist oversight. Our results support adoption of such side-aware constraints before routine deployment. Breast density influenced both artificial intelligence and reader performance. Sensitivity decreased in heterogeneously and extremely dense categories, consistent with lesion masking from X-ray attenuation and reduced conspicuity [1,4,27]. Studies that integrate prior examinations, tomosynthesis, or targeted ultrasound have documented partial recovery of sensitivity, suggesting that artificial intelligence systems which fuse prior or cross-modality context may mitigate density-related loss [6,17,29]. Until such fusion is widely available, cautious interpretation in dense breasts and targeted secondary imaging remain necessary.

Comparison with published benchmarks indicates that our radiologist accuracy aligns with contemporary single-reader performance, while the artificial intelligence profile falls within the range reported for research-grade systems evaluated outside the original development distribution [2,11,17,24]. Importantly, several multicenter studies have shown that artificial intelligence specificity is most unstable when prevalence, vendor mix, and positioning differ from training data [9,14,30,33,34]. This reinforces the value of local calibration prior to adoption.

As an operational model, a concurrent-aid approach elevates higher-risk studies in the worklist and cues focused assessment yet maintains independent judgment [5,15,20,35,36]. To reduce benign overcalls, some programs apply decision curves that trade small losses in sensitivity for meaningful reductions in unnecessary recalls [2,24,37]. Artificial intelligence as an independent second reader may substitute for double reading in resource-constrained settings only after specificity and laterality accuracy are improved and clear escalation rules for discordant cases are validated prospectively [4,22,30]. User interface design remains critical to avoid automation bias and to protect the benefits of independent reads [10,19].

Safety and governance require noting that, while laboratory anchoring reduces verification bias for malignancy, benign ground truth still partly depends on imaging stability, which can skew specificity estimates [1,35,38]. Prospective quality assurance should include periodic review of false positives and false negatives with feedback loops to both the model and readers, an approach associated with measurable gains in other imaging domains [18,39,40]. Transparent reporting with confidence intervals, calibration plots, and decision curves enables oversight bodies to judge tradeoffs that reflect local priorities [2,29,41].

Equity and generalizability require attention, as documented performance drift across vendors, compression paddles, and population subgroups can affect both sensitivity and specificity [9,14,30]. In addition, broader implementation of AI in breast imaging must consider the possibility of uneven performance in underrepresented populations, which could amplify existing disparities if not explicitly evaluated during external validation. Privacy and governance also remain important, particularly when AI systems are developed or adapted using clinical imaging and report data [42]. Although the present study used de-identified data under institutional oversight, broader questions related to data privacy, data stewardship, and responsible AI development warrant continued attention. External validation across diverse sites with predefined acceptability margins and periodic recalibration after deployment is therefore essential [17,30,31], including adherence to reporting standards, public documentation of thresholds and updated cadence support reproducibility, and fair comparison across systems [19,29]. Implications for clinical practice: Given its higher sensitivity but lower specificity, artificial intelligence is best used to trigger second looks on studies otherwise read as negative, helping reduce missed cancers [5,10]. However, because benign overcalls carry cost and anxiety, programs should track recall rate, positive predictive value of biopsy recommendation, and time to diagnostic resolution after adoption [2,22,24]. The feature type profile suggests that readers should be most skeptical of artificial intelligence prompts for mass in dense tissue and for subtle calcifications, while considering artificial intelligence prompts for diffuse parenchymal patterns as higher-yield [8,13,18].

To boost specificity, augment training sets with common benign look-alikes including tissue overlap, fibroadenomas with calcifications, and postsurgical change while employing cost-sensitive losses that penalize false positives [8,12,19]. Second, improve calcification detection by increasing native pixel resolution, adding calcification-specific augmentations, and using multi-scale heads tuned for small object detection [8,23,27]. Third, harden laterality by enforcing side-aware constraints and cross-view agreement and by rejecting outputs that conflict with view geometry [3,16,26,31]. Fourth, explore fusion of priors and tomosynthesis to mitigate density effects and improve robustness across acquisition variability [6,17,29,30].

All 100 examinations included in the analytic test set underwent biopsy and had histopathologic confirmation. The present analysis was therefore restricted to these biopsy-verified cases to ensure a reliable pathology-based reference standard. At the same time, the biopsy-confirmed design allowed direct comparison of radiologist and AI performance against histopathology as the gold standard. Although this strengthens internal validity, it also enriches malignancy prevalence, and the resulting specificity, accuracy, and overall performance metrics are therefore not intended to represent real-world screening performance or to generalize directly to lower-prevalence populations. Prospective studies in broader, unselected screening cohorts will be necessary to assess true clinical utility, operating characteristics, and downstream workflow impact under routine practice conditions.

Future prospective studies should also report the number of participating radiologists, their breast-imaging experience, and whether interpretations are based on single-reader or consensus workflows, as these factors may influence comparative performance. The next step is prospective, workflow-integrated validation across diverse acquisition settings, with pre-specified operating thresholds and monitoring of patient-centered and system outcomes (e.g., recall rate, biopsy yield, time to diagnostic resolution). Future studies should also include head-to-head comparisons with other reasoning-capable and multimodal LLMs, such as Gemini, DeepSeek, and Gemma, as well as established mammography-specific AI systems, to determine whether the performance trade-offs observed here are model-specific or reflect broader limitations of current AI approaches in biopsy-referred mammography. With careful calibration, side-aware constraints, and targeted improvements in calcification handling and false-positive control, such systems may ultimately contribute to earlier cancer detection while preserving trust, transparency, and patient well-being.

## 5. Conclusions

Overall, ChatGPT-5.2 Thinking demonstrated higher sensitivity than radiologists for detecting biopsy-confirmed malignancy. This, however, was accompanied by a substantial reduction in specificity and a higher false-positive burden, yielding lower overall accuracy. Taken together, the observed trade-off supports positioning this system as decision support, most plausibly as a concurrent “second-look” cue or triage signal, rather than as a replacement for radiologist interpretation. These findings should nonetheless be interpreted with caution given the small sample size and the prevalence-enriched biopsy-referred design, which may limit generalizability to broader screening populations.

Performance heterogeneity across radiologic feature types provides additional, clinically actionable context. The model showed comparatively better performance with broader parenchymal density patterns, but it was less reliable for microcalcifications and prone to overcalling mass-like findings, which likely contributed to benign over-referral. Moreover, laterality agreement was only moderate, highlighting a safety-relevant limitation because incorrect side localization can increase downstream imaging, delay resolution, and heighten patient distress even when malignancy is correctly suspected. These results emphasize that real-world utility will depend not only on case-level discrimination but also on reliable localization and workflow-aware presentation that minimizes unnecessary escalation.

## Figures and Tables

**Table 1:** Confusion matrix results and performance of radiologists and ChatGPT-5.2 Thinking compared with the biopsy-confirmed gold standard in the biopsy-referred cohort.

	Gold Standard Positive	Gold Standard Negative
Radiologist Positive	TP = 50	FP = 17
Radiologist Negative	FN = 11	TN = 22
AI Positive	TP = 58	FP = 35
AI Negative	FN = 3	TN = 4

**Table 2:** Sign-specific diagnostic performance of ChatGPT-5.2 Thinking.

Sign	TP	TN	FN	FP	Sensitivity	Specificity	Accuracy	Precision
MASS	15	5	6	35	0.714	0.125	0.333	0.3
AS	5	42	6	8	0.455	0.84	0.783	0.385
MC	4	30	12	15	0.250	0.667	0.605	0.211
PD	9	16	2	34	0.818	0.32	0.417	0.209

## Data Availability

The data presented in this study are not publicly available due to institutional restrictions and data governance policies. De-identified data may be made available from the corresponding author upon reasonable request and with appropriate approvals.

## Abbreviations

The following abbreviations are used in this manuscript:

AI	Artificial intelligence
BI-RADS	Breast Imaging Reporting and Data System
CC	Craniocaudal
MLO	Mediolateral oblique
TP	True positive
TN	True negative
FP	False positive
FN	False negative
AS	Asymmetry
MC	Microcalcifications
PD	Parenchymal distortion

## Appendix A

Exact prompt used:

“Read the uploaded BI-RADS^®^ 5th Edition guide (PDF) carefully, learn it fully, and master its diagnostic criteria. You will be presented with breast imaging cases. Use only the uploaded BI-RADS guide for evaluation—do not use any external sources. The analysis results must include the following:

BI-RADS Suspicion Score

Assign a score from 0 to 5 according to BI-RADS^®^ 5th Edition criteria.

Radiological Signs

Use standardized BI-RADS^®^ descriptors based on findings in the case:

- mass: Mass
- mc: Microcalcifications
- as: Asymmetry
- pd: Parenchymal Distortion
- Combinations are allowed (e.g., as/mc, mass/pd, etc.)

Cancer Side

Indicate whether the lesion is on the Left (L) or Right (R) breast based on your findings.”
